# Growth hormone: isoforms, clinical aspects and assays interference

**DOI:** 10.1186/s40842-018-0068-1

**Published:** 2018-08-28

**Authors:** Júnia Ribeiro de Oliveira Longo Schweizer, Antônio Ribeiro-Oliveira Jr, Martin Bidlingmaier

**Affiliations:** 1 Endocrinology Laboratory of Federal University of Minas Gerais. Alfredo Balena, 190, Santa Efigênia, Belo Horizonte, 30130-100 Brazil; 2 Endocrine Laboratory, Medizinische Klinik und Poliklinik IV, Klinikum der Universität München, Ziemssenstraße 1, 80336 Munich, Germany

**Keywords:** Growth hormone, Growth hormone isoform, Growth hormone molecule, 22 k growth hormone isoform, 20 k growth hormone isoform, Acromegaly, Growth hormone deficiency, Growth hormone assays, IRP 98/574

## Abstract

The measurement of circulating concentrations of growth hormone (GH) is an indispensable tool in the diagnosis of both GH deficiency and GH excess. GH is a heterogeneous protein composed of several molecular isoforms, but the physiological role of these different isoforms has not yet been fully understood. The 22KD GH (22 K-GH) is the main isoform in circulation, followed by 20KD GH (20 K-GH) and other rare isoforms. Studies have been performed to better understand the biological actions of the different isoforms as well as their importance in pathological conditions. Generally, the non-22 K- and 20 K-GH isoforms are secreted in parallel to 22 K-GH, and only very moderate changes in the ratio between isoforms have been described in some pituitary tumors or during exercise. Therefore, in a diagnostic approach, concentrations of 22 K-GH accurately reflect total GH secretion. On the other hand, the differential recognition of GH isoforms by different GH immunoassays used in clinical routine contributes to the known discrepancy in results from different GH assays. This makes the application of uniform decision limits problematic. Therefore, the worldwide efforts to standardize GH assays include the recommendation to use 22 K-GH specific GH assays calibrated against the pure 22 K-GH reference preparation 98/574. Adoption of this recommendation might lead to improvement in diagnosis and follow-up of pathological conditions, and facilitate the comparison of results from different laboratories.

## Background

The measurement of circulating concentrations of growth hormone (GH) is an indispensable tool in the diagnosis of both GH deficiency and GH excess. GH is a heterogeneous protein composed of several molecular isoforms, but the physiological role of the different isoforms has not yet been clarified. Owing to the fact that assays to specifically measure the different GH isoforms are not easily available, only a limited number of studies have investigated them under various clinical conditions. Most commercially available GH assays have not been fully characterized with respect to their cross-reactivity with the different isoforms. It must be assumed that most assays measure a mixture of isoforms with differences in affinity. This in part explains why despite some advances in design, practicability and sensitivity of the assays, discrepancies between GH concentrations reported from different GH assays increased over the last decades [1–3]. This article reviews available information on the main GH isoforms as well as their impact on GH measurements in clinical practice.

## GH molecule

Growth hormone belongs to a superfamily of cytokines, which includes interleukins, cytokines, as well as leukemic, neurotropic, and growth factors [4]. GH is a polypeptide hormone exhibiting molecular heterogeneity. It consists of a complex mixture of molecular isoforms and their multimers. In humans, the genetic locus that codes GH resides on chromosome 17q24.2 [4, 5], has 46.83 kilobases and contains five GH related genes. These multiple genes most likely arose from gene duplication. Each of these genes is composed of five exons and four introns: GH1 (or GH-N), GH2 (or GH-V), CS1, CS2 and CSL. While the GH1 is mainly expressed in somatotropes of the pituitary gland, the GH2 and CS (chorionic somatomammotropin also known as placental lactogen) are expressed exclusively by the placenta in females during pregnancy. The CSL (CS-like protein) is expressed at low levels and its function remains unclear [6].

### Pituitary GH - isoforms and fragments

#### 22 K-GH

**Fig. 1:**
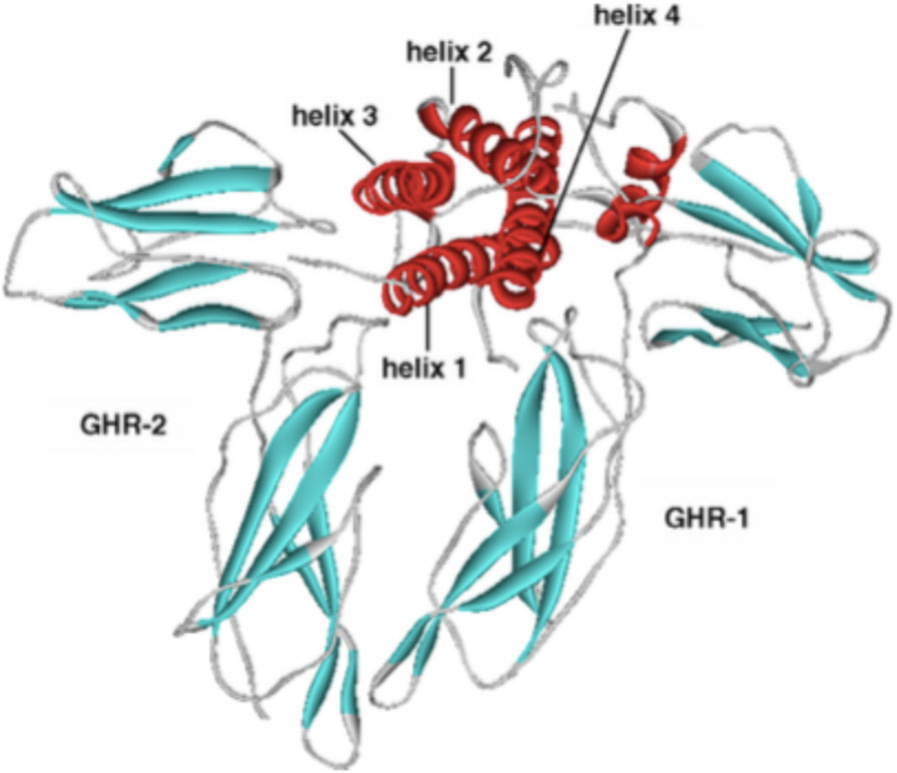
The 22 K-GH isoform structure and GH-R (GH receptor) biding sites (reproduced with permission from [19]

The GH1 is expressed mainly in somatotrope cells of the pituitary gland. Expression in the immune system has been described, although there is no evidence that lymphocyte derived GH significantly contributes to circulating GH concentrations. The main product of this gene is a 191 amino acids single-chain protein stabilized by two disulphide bridges. The molecular weight is 22,129 Da [7]. This 22 K-GH molecule is the main GH isoform, representing more than 90% of total GH in circulation. Its tertiary structure is a 4-helical (Fig. 1) twisted bundle with unusual connectivity. The helices run up-up-down-down instead of the more usual up-down-up-down form. There are two binding-sites interacting with the GH receptor, namely site I and site II. Spectrums of posttranslational modifications of this isoform, including acetylation, phosphorylation, deamidation and glycosylation have been described, which can potentially modify GH actions [8, 9]. GH is best known from its growth promoting activity in children, but also has important biological activities in adults. These include lipolysis, glucose-, calcium- and phosphorous-metabolism as well as lactogenesis and immune function. The previous knowledge that some hormones and cleaved fragments have biological actions, like POMC (proopiomelanocortin), ACTH (adrenocorticotrophic hormone) and β-endorphin, as well as ANP (atrial natriuretic peptide) and BNP (brain natriuretic peptide), has led to the suspicion of GH isoforms existence and possible functions.

#### 20 K-GH

**Fig. 2:**
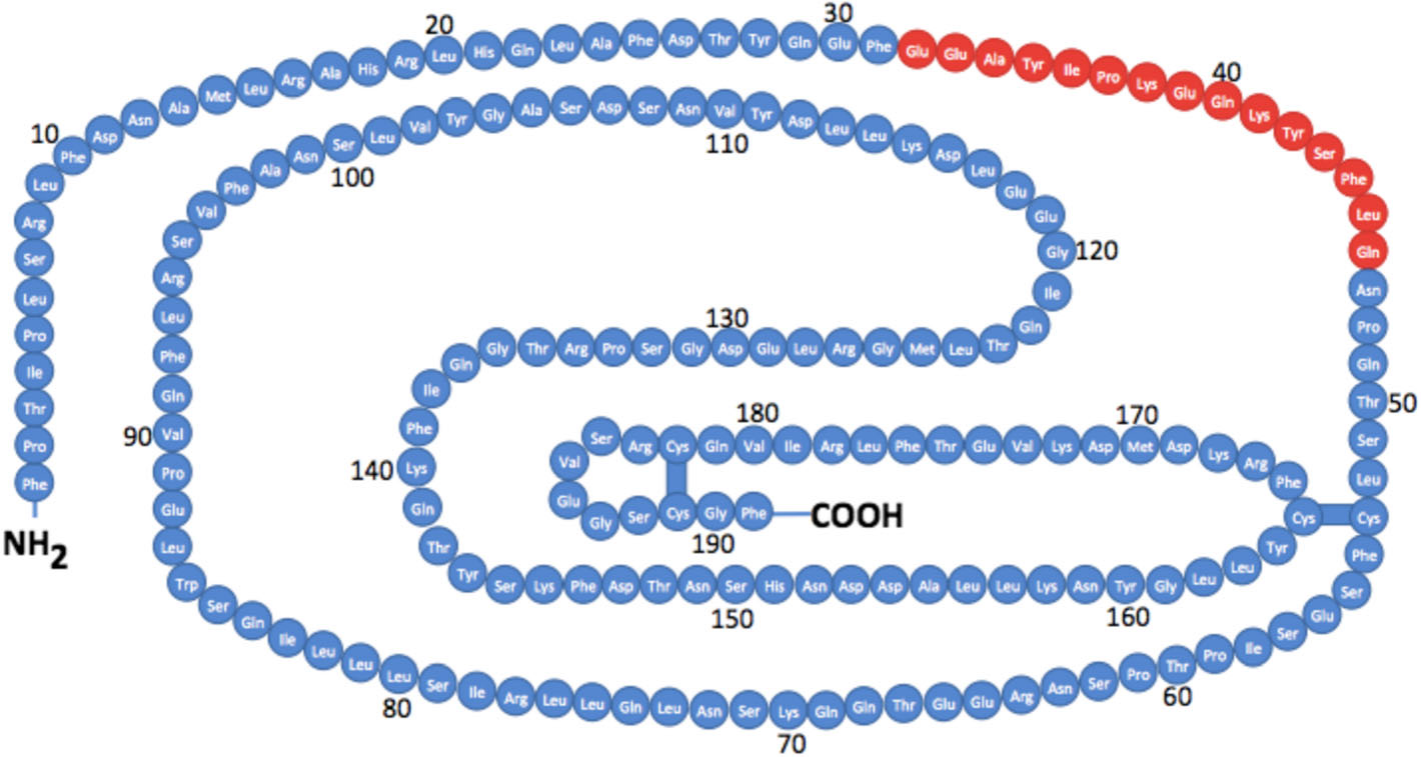
The 22 K-GH isoform molecule with 191 residues. The 20 K-GH isoform has the same residues, except the 32–46 residues, which are missing

The second most abundant GH isoform is the 20 K-GH molecule. It is derived from GH-1 by alternative pre-messenger ribonucleic acid (pre mRNA) splicing of exon 3. The structure is similar to 22 K-GH except for a deletion of the internal residues 32–46 (Fig. 2). Therefore, 20 K-GH consists of 176 amino acids only. The molecular weight is 20,274 Da. This smaller GH isoform represents about 10% of total circulating GH. There is controversy in the literature regarding the biological function of 20 K-GH [10–13]. Most in vitro and animal studies report similar activities to promote growth and stimulate lipolysis, while some authors discussed reduced diabetogenic and anti-natriuretic activities [14–17]. The 20 K-GH isoform is more prone to dimerize, leading to slower clearance when compared to 22 K-GH. This potentially could lead to a change in the relative abundance of 20 K- and 22 K-GH isoforms in plasma over time following a secretory burst and possibly an extended action of the 20 K-GH isoform. Since the residues 32–46 missing in the 20 K-GH isoform overlap with the GH receptor binding site 1 (Figs. 1 and 2), the strength of interaction with the GH receptor could be different from the 22 K-GH [14]. However, there is compelling evidence that both 22 K- and 20 K-GH can activate Janus Kinase 2 (JAK2), signal transducers and activators of transcription 1, 3 and 5 (STATs 1/3/5), although the level of STAT 1/3/5 phosphorylation induced by 22 K-GH are higher than those of 20 K-GH [18].

### GH fragments

As aforementioned, although the 22 K-GH is the main predicted protein product from the GH gene, posttranscriptional and posttranslational processing can lead to different GH isoforms. A number of such smaller fragments or isoforms has been described, but not all of them could be independently confirmed [10, 19]. It is important to realize that methodological differences in the experimental approach to identify these fragments as well as differences between species or matrices might explain the inconsistency of some findings. It has been reported that a single specific cleavage can generate two contiguous GH fragments of 5 K (fragment 1–43) and 17 K (fragment 44–191). These isoforms were synthesized and isolated from pituitary extract but have also been reported to occur in significant amounts in circulation. It has been suggested that 5 K-GH has similarity with the GH N-terminal region, has insulin-like activity and can be an in-vitro substrate of dipeptidyl peptidase [20]. Independently, a polypeptide of 17.5 K resulting from GH exon 3 skipping has been described in pituitary and circulation, representing 1 to 5% of GH transcripts. It reportedly exists mainly under pathological conditions. However, identity to the above mentioned 17 K-GH fragment 44–191 could not be demonstrated by the assay used [21]. Another synthetic peptide obtained by stop sense molecular inclusion in the GH gene sequence (fragment 53–134) was also discussed to correspond to 17 K-GH isoform. Other molecular variants including 12 K-GH, glycosylated GH, deamidated GH, and phosphorylated GH have also been described. However, more research is obviously needed to fully understand the nature of the smaller isoforms [7, 22–24].

At the other end of the molecular weight spectrum, larger forms of GH molecules can be found: 35 K-GH and 45 K-GH, as well as GH homo- and hetero-dimers and -oligomers have been found [10, 19]. It is still uncertain whether these isoforms could have the same biological actions potency of the 22 K-GH. They may act as either 22 K-GH agonists or antagonists, thus regulating biological functions of GH.

In addition to naturally occurring GH isoforms, additional isoforms and fragments have been synthesized. Currently, the GH receptor antagonist pegvisomant is the most relevant example of an artificial GH isoform. The amino acid sequence differs from that of naturally occurring 22 K-GH in only eight amino acids in binding site 1 (H18D, H21N, R167N, K168A, D171S, K172R, E174S, I179T), and one amino acid in binding site 2 (G120R). Also, pegylated residues were added to Lys residues in the 22KD-GH, producing a molecule with 42-46 K. These targeted mutations led to the generation of a GH molecule which can bind to one GH receptor molecule by very high affinity but does not correctly bind to the other molecule of the dimeric GH receptor. This prevents signal transduction, making the molecule a potent GH antagonist used to the treatment of acromegaly [25–27].

Among the smaller GH molecules, which have been synthesized is GH 30–54 [19]. It contains the segment GH32–46, which distinguishes the two main GH isoforms, i.e. the 22 K- and 20 K-GH. Its presence in biological fluid and the biological effects are not known. Another fragment, GH 108–129, may be originated from natural biosynthesis or GH fragmentation. It encompasses the heli× 3 and site-II regions, the latter related to GH receptor interaction. Since mutations in this site could result in GH receptor binding interference and ultimately in growth disruption, this peptide could potentially serve as another GH antagonist and has been patented. A fragment 147–191 is obtained by enzymatic proteolysis and may be responsible for the generation of the fragment 177–191 (AOD9401). This late fragment is a short peptide, and one group has claimed it has GH like activity and the potential for treating obesity, due to its action on lipolysis [28, 29]. Interestingly, many of the fragments first were theoretically hypothesized, and then produced later on [30]. However, the significance of all these products for routine treatment remains unclear.

### Placental GH

The placental GH (GH2 or GH-V) shares similarity with GH1 gene, except for 13 residues difference. It contains a consensus sequence for N-glycosylation at position 140, suggesting the existence of two GH2 isoforms: a glycosylated and a non-glycosylated form. The placental GH is a more basic protein, expressed exclusively in syncytiotrophoblast of the placenta. It is progressively released into the maternal circulation with peak concentrations reached in late pregnancy. Due to its high somatogenic activity, IGF-I concentrations in pregnant females tend to increase. In turn, these increasing IGF-I concentrations lead to an almost complete suppression of the pituitary GH-N secretion with advancing pregnancy. Although there is evidence of 20 K-GH isoform derived from placental GH, this gene is less prone of alternative splicing and does not represent the major origin of this isoform [31]. There is also evidence that placental derived 20 K-GH has lower diabetogenic and lactogenic activities when compared to the placental 22 K-GH [32]. Chorionic somatomammotropin (CS), the other important product of the GH gene family expressed in the placenta, has 85% structural homology with pituitary GH but does not have important somatogenic bioactivity. GH-V and CS will not be further discussed herein, as their importance are limited to the gestational period, which is not an objective of this article.

## GH isoform secretion in healthy and disease

### Physiology

22 K-GH is secreted from the pituitary gland under hypothalamic control. Secretion is classically stimulated by GHRH and inhibited by somatostatin. 22 K-GH secretory pulses occur every 2–3 h with great amplitude variance. The largest pulses usually occur at night, during slow wave sleep [33]. The 22 K-GH secretion pattern may differ between sexes, mainly due to estrogenic effect in females. Females usually have higher secretion rates, higher interpeak levels and more erratic secretion patterns of this isoform than males [34]. The 20 K-GH isoform has been reported to be higher in females than males, although the ratio of 20- to 22 K-GH usually does not differ between sexes [35, 36]. 22 K-GH secretion patterns also change with age. The 22 K-GH peaks are higher in puberty, and secretion rates decrease later in life by approximately 15% per decade. Other factors also affect GH secretion: Obesity is known to attenuate GH secretion whereas physical activity, stress and fasting are acute stimuli. In physiological state, the 20 K-GH and the 22 K-GH isoforms are secreted in a pulsatile manner, in a constant molar ratio and the peaks from both isoforms were coincident in healthy individuals, although much lower for 20 K-GH isoform [36].

Although there are other sites of GH clearance (e.g. the liver), the major portion of the metabolic clearance of monomeric GH occurs in the kidney. There is an efficient glomerular filtration and degradation in the proximal tubule. The urinary GH corresponds to only 1/10,000th of glomerular filtered GH. Some studies reported differences in clearance of isoforms, with a slower clearance for oligomeric GH and 20 K-GH compared to the main 22 k isoform. One study demonstrated an approximately 30% reduced clearance for 20 K-GH [37], although direct comparison of results from isoform specific assays might be difficult. There is little information about salivary GH, but in normal subjects it seems to be 1000-fold lower than serum GH. Since both, urinary and salivary GH are present in much smaller quantities than serum GH, methods to assess GH in these matrices are difficult and less standardized. Urinary GH has also been shown to be less stable than serum GH. Thus, it is no surprise the main matrix to measure 22 K- and 20 K-GH, and also the main source of our knowledge about GH isoforms is serum.

### Exercise

Exercise is a well-recognised condition that naturally stimulates GHRH and GH release in the circulation [38]. It can alter nocturnal GH secretion pattern by attenuating burst mass and amplitude, but increasing burst frequency. Therefore, the total nocturnal GH secretion is not altered [39]. Exercise can increase lactate acid, and there is a relation between GH secretion and blood lactate [39].

Some GH isoform studies measured various GH isoforms with distinct methods under different physical activity protocols. Wallace et al. [40] showed that all GH isoforms (22 K-, 20 K- and non-22 K-GH isoforms) increased during acute exercise. The 22 K-GH (polyclonal immunoradiometric assay) was the main GH isoform produced during physical activity and peaked at 30 min. The 20 K-GH (ELISA) and the non 22 K-GH measured by the 22 K-GH exclusion assay exhibited a somewhat greater increase during the post exercise period. This temporarily increased the relative abundance of the non-22 K-GH isoforms. The authors suggested that these isoforms might play a role in preventing post-training hypoglycemia, as the GH isoforms could have diabetogenic effects. Another study [41] found a moderate modulation of GH isoforms after acute exercise. As expected, concentrations of 22 K-GH (IFMA) and 20 K-GH (specific ELISA) isoforms increased, but – in contrast to the above mentioned study – the increase was greater for 22 K-GH. Accordingly, in this study the ratio 22 K-/20 K-GH was slightly increased for a short period after acute physical activity. After chronic resistance exercise, most studies did not find major alteration in different molecular weight GH isoforms [42]. However, in a study by Pierce et al. [43] it was shown that acute and chronic resistance exercise led to the appearance of similar amounts of disulphide-linked GH aggregates. The physiological significance of this alteration is unknown. Some studies used a very different analytical approach to study different molecular weight GH isoforms in physical activity. In these studies, GH isoforms were separated into categories greater than 60 K (> 60 K), 30-60 K and less than 30 K (< 30 K). In females, acute heavy resistance exercise led to an increase in 30-60 k and > 60 k, but not in the < 30 K molecular weight GH [44]. Before exercise, stronger women had greater total GH than the weaker ones, while the latter had higher smaller weight GH fractions (< 30 K). All GH isoforms increased after exercise in both, strong and weak untrained women, although the lower molecular weight variants were less responsive to greater amounts of exercise in stronger women [45]. The use of oral contraceptive (OC) in untrained women also seemed to influence the GH response during exercise when assessed by these assay methods, with higher abundance of high molecular weight GH in both resting and post exercise states in the OC group [46]. In contrast, other studies only confirmed that in the basal state all GH isoforms tend to be higher in females, but the 20 K–22 K-GH ratio was not different between sexes. In addition, oral contraceptive administration to postmenopausal women and testosterone administration to hypogonadal men also led to an increase in both 20 K- and 22 K-GH isoforms, but did not alter the 20-to-22 K-GH ratio [36]. The latter study used specific immunoassays to measure the 20 K- and 22 K-isoforms separately.

In summary, though the exercise induced increase in GH is known since a long time and has convincingly been demonstrated by several groups, any potential alterations in the relative abundance of the different isoforms have never been uniformly shown across studies. The use of different in-house research-type assay methodologies to measure concentrations of isoforms makes it very difficult directly comparing results from different studies. However, regardless of the assay methods used, the changes in isoform composition with exercise – if any - were small and of short duration.

### Doping

Although evidence supporting performance enhancing effects in healthy trained subjects are lacking, it is known that GH is abused by some athletes [47]. For this reason, the World Anti-Doping Agency (WADA) classifies GH as a prohibited substance. Detection of GH doping, however, was considered I possible for a long time because recombinant GH is identical in its amino acid sequence and physicochemical properties to the main isoform of endogenous GH. The pulsatile secretion pattern of GH also makes it impossible to use high serum GH levels as evidence for exogenous GH administration. The improved understanding of the physiology of GH isoforms and their regulation by the administration of the other GH isoforms, however, has helped to develop a valid doping test. It has been shown that administration of the 20 K-GH isoform reduces secretion of the 22 K-GH isoform levels [48]. In turn, administration of exogenous recombinant 22 K-GH administration rapidly suppresses non-22 K- and 20 K-GH isoforms. Isoforms other than 22 K-GH remain low for approximately 24 h, coupled to a reduction in the 20 K-to-22 K-GH ratio [36, 49, 50]. This knowledge enabled the development of the so-called GH isoform test as one WADA’s recommended anti-doping tests [51, 52]. Its advantage is the direct detection of the molecular changes induced by the administration of recombinant GH, although the short time window limits its use to the first 12-36 h after recombinant GH administration.

### Pathological conditions

#### Acromegaly

In acromegaly, the rhythmicity of GH secretion seems to be preserved [34]. There were some studies evaluating the GH isoforms in acromegaly in the past decades, although the studies used different methods and measured different isoform fractions. Boguszewski et al. [53] evaluated the “non-22 K-GH isoform” in men with acromegaly before and 1 year after transsphenoidal surgery. The relative abundance of the non-22 K-GH isoform was increased in active acromegaly when compared to inactive acromegaly and healthy controls. Interestingly, the proportion of non-22 K-GH isoform in active acromegaly remained high after non-curative surgery, while patients with controlled acromegaly achieved a percentage of the non-22 K-GH isoform similar to healthy individuals. Tsushima et al. [54] for the first time studied the 20 K-GH isoform in acromegaly (by ELISA). They showed an increase of this isoform in active acromegaly, and also an increased 20- to 22 K-GH ratio in acromegaly compared to healthy controls. Another group [36] studied GH secretion pattern in acromegaly and healthy controls by measuring 20 K- and 22 K-GH every 20 min for 24 h. Although there was an increase in 20 K-GH isoform in patients with acromegaly, this isoform increased in parallel with 22 K-GH, keeping the 20- to 22 K-GH ratio very similar to the ratio seen in healthy controls. There is also conflicting data regarding GH isoforms after somatostatin analogue treatment in patients with acromegaly. Although Murakami et al. did not find a modification in the 20- to 22 K-GH ratio after acute octreotide treatment [55], Leung et al. [36] described a rapid reduction in both isoforms, but with a relative increased in the 20- to 22 K-GH isoform ratio. The authors speculate that this was caused by a shorter half-life and thus a faster decrease in the 22 K-GH isoform when compared to the 20 K-GH isoform. A more recent study [56] evaluated patients with acromegaly before and after 6 months of octreotide LAR treatment. The 20 K- and 22 K-GH were increased in patients with acromegaly when compared to healthy controls, but the 20- to 22 K-GH isoform proportion did not change. Furthermore, this study did not find alterations in the 20 K- to 22 K-GH ratio before and 6 months after initiation of octreotide treatment. A limitation of this study was that only 13% (3/23) of acromegaly patients were controlled after octreotide treatment, precluding final conclusions about potential changes in the 20 K- to 22 K-GH ratio related to octreotide therapy.

#### GH deficiency

GH deficiency obviously more likely is associated with very low GH concentrations. The very low concentrations provide a limitation to the study of GH isoforms, since the smaller, less abundant isoforms (eg. 20 K-GH) are even lower and commonly below the detection limit of most assays. Accordingly, there are only very few studies on GH isoform secretion in the GH deficiency field, and these studies also differ considerably in the analytical methods used. To overcome the problem, some of the studies used various stimulation test protocols to increase GH concentrations and make the isoforms accessible to existing analytical methods. Some studies reported no influence of age, pubertal stage and sex on the 20 K/22 K-GH ratio in normal and GH deficient children and adults. There was also no change in 24-h GH secretion pattern after arginine and hypoglycemia stimulation tests [17, 35, 36]. Another group studied normal children after a different stimulation test (GHRH). They found that both, the non-22 K-GH and 22 K-GH isoforms increased after GHRH administration, but no change in the ratio. After a second GHRH stimulus, patients who still responded with a GH peak secretion greater than 10 ng/mL had lower non-22 K-GH levels than non-responders. These data could suggest isoform related differences in the recovery of somatotrope function or differences in GH isoforms metabolic clearance, but also could be related to assay sensitivity [57]. Pagani et al. [41] studied 22 K- and 20 K-GH isoforms in GH deficient patients before and after several pharmacologic stimuli such as arginine, L-dopa or glucagon. They found a significant increase in both 22- and 20 K-GH isoforms, but describe a slight increase in the 22 K-/20 K-GH ratio. The 22 K-GH was the most abundant isoform even in a state of reduced GH secretion. As discussed above the use of different assays to assess GH isoforms could explain the difference in results. Furthermore, the groups studied different stimuli during dynamic tests.

#### Prader-Willi syndrome

Prader-Willi syndrome (PW) is a complex disorder associated to hypogonadism, behavioural and cognitive impairment, alongside with obesity and short stature. The GH secretion is abnormal, but the etiology is unknown. One study has evaluated GH isoforms in obese and non-obese, and GH deficient and non-GH deficient patients with PW submitted to GHRH plus arginine test [58]. There was no difference in the 22 K- and 20 K-GH isoform ratio at baseline between obese and non-obese patients. The stimulation test increased 22 K- and 20 K-GH isoforms in non-obese and non-GH deficient PW patients. The GH response for both isoforms was higher in non-obese PW patients and there was no difference in isoforms between GH deficient and non-GH deficient in these non-obese PW patients. The ratio of circulating levels of 22 K-to-20 K-GH did not alter during the test in all studied groups. This study shows that alteration in GH isoforms generation may not be implicated in etiology of GH pattern alteration in PW [58]. Furthermore, this group compared GHRH plus arginine (GHRH+ARG) stimulation test with arginine (ARG) only in PW. Although both tests increased 20 K-GH peak, it was higher in GHRH+ARG than in ARG group. This study further confirmed absence of alterations in the 20/22 K-GH ratio in both stimulation tests [59].

#### Other conditions

Little data exist regarding GH isoforms in other conditions. One study showed that anorexia was associated with a higher abundance of the 20 K-GH isoform (20 K/20 K-GH + 22 K-GH), but there was no difference in hypothyroidism, hyperthyroidism and non-insulin dependent diabetes [35].

## Impact of molecular heterogeneity on GH measurement in clinical routine

The study of GH isoforms in physiology and in pathologic states was only possible with the development of more sensitive, isoform specific assays. Historically, GH has been measured by a wide spectrum of different analytical methods ranging from bioassays to radio receptor assays, immunoassays and mass spectrometry approaches. For a long time, isoform specificity on most methods was unknown, and for many assays used in clinical routine it is still unknown. Cell based and radio receptor assays do not distinguish specifically between isoforms, while mass spectrometry assays today still lack sensitivity particularly to measure the less abundant isoforms. Mass spectrometry assays also not used in clinical routine. The most commonly used method to assess GH concentrations in clinical routine are antibody-based immunoassays. Theoretically, detailed investigation of the epitopes recognized by the antibodies used in these assays would allow characterization of each assays isoform specificity. In fact, most of the studies on regulation of GH isoforms reviewed above have used some well-characterized isoform specific immunoassays. However, for GH assays used in high throughput routine laboratories the extra effort to characterize the isoform specificity of the assays is rarely done. However, it is important to be aware that each of this routine GH immunoassay – depending on the antibodies used - will pick a different spectrum of total GH isoforms. Many factors potentially affecting comparability of GH immunoassay have been described. These include nature and composition of the assay calibrator, interference from the growth hormone binding protein (GHBP) and also matrix effects. However, the differential recognition of GH isoforms remains one of the key problems of assay comparability. It inherently leads to issues regarding the quantification of GH and contributes to the known discrepancies between GH concentrations in a given sample obtained from measurements by different assays. Generally, older assays based on polyclonal antibodies commonly measured a broader isoform spectrum. The advent of monoclonal antibody assays brought more specificity to one or few of GH isoforms. It has been described that this increased specificity led to greater differences between the absolute concentrations reported from assays using different antibodies. Because of the recognition of only a certain spectrum of isoforms, the newer, monoclonal antibody based assays also have a tendency to report lower GH concentrations compared to older assays using polyclonal antisera. This is important to keep in mind when applying cut-offs from guidelines to the interpretation of GH data: If cut-offs were established by polyclonal assays, but in clinical routine today monoclonal antibody based assays are in use, the cut-offs might have to be adapted to reflect the lower GH concentrations reported by modern GH assays.

To facilitate the uniform adoption of cut-offs from guidelines continued efforts have been undertaken to harmonize results from different immunoassays for GH. General recommendations for performance characteristics of ideal GH assays have been published by scientific societies to guide physicians working in the field [1–3].

### Isoform specificity for common assays

The existence of GH isoforms affects the comparability of GH assays in two ways: As described above, different assays recognize different isoforms. In addition to this, different reference preparations are in use to calibrate the GH assays – the older ones consisting of a mixture of pituitary GH isoforms, the newer ones – of recombinant origin – consisting of the 22 K-GH isoform only. In an immunoassay, the analyte concentration is determined by comparing the signal generated in the sample to a signal from samples with known amounts of the analyte. Consequently, the preparation used for GH assay standard curve has an important impact on GH measurement and results. In the past, the GH assays were done with pituitary extracts, and international reference preparations (IRP) were 66/217 and 80/505. Both contained a variety of GH isoforms and the exact amount of GH was unknown. The concentrations were arbitrarily assigned as 2.0 and 2.6 U/mg for IRP 66/217 and 80/505, respectively. Subsequently, new reference preparations were produced by recombinant technologies. These reference preparations consist of the 22 K-GH exclusively (IRP 88/624 and 98/574). Now it became possible to make GH assays traceable to a mass unit of the IRP 88/624 (micrograms per liter). For historic reasons, a conventional unit was also assigned (3.0 U/mg), though recent guidelines do no longer support the use of conventional units. Recently, the next generation of the recombinant international IRP has been introduced (98/574). Basically identical to 88/624, the new preparation is of high purity (> 96% 22 K-GH) and shows adequate stability, bioactivity and availability [2].

Table 1 lists a number of GH immunoassays commonly used in clinical routine. All these assays meanwhile are calibrated to the recombinant IRP 98/574. Though the use of a common calibrator has slightly improved assay agreement over the last years [60], there is still considerable disagreement between GH assay results, and the disagreement still has considerable impact on the diagnosis of GH related disorders [61]. For many of the assays information on isoform specificity of the antibodies is not available or incomplete. One of the assays with published characterization of the antibodies (IDS) does not cross-react with an artificial GH isoform: pegvisomant is a mutated GH molecule, which is used as a drug to treat GH excess (acromegaly) by blocking the GH receptor. Most other available GH assays cross-react with pegvisomant, leading to falsely high or low results depending on antibody specificity and assay design.

**Table 1:** Characteristics of commonly used commercial assays for GH (according to manufacturers instructions/kit inserts available to the authors or according to published data). Calibration has changed for several assays in recent years, and the process is ongoing. To the best of the authors knowledge the assays listed here have uniformly adopted the latest recombinant standard for all countries. The list of assays is not complete. Additional hGH assays exist, including an unknown number of in-house assays. (modified from [3]

Manufacturer	Name	Assay principle	Calibration	Isoform-specificity	Measuring range	Recommended sample material	Comment
Siemens	Immulite 2000	Two-site Chemilumminescent immunometric assay	98/574	Not provided	0.05 to 40 ng/mL Analytical sensitivity: 0.01 ng/mL	Serum	ng/mL × 3.0 ➔mIU/L
Diasorin	Liaison hGH	Chemiluminescent sandwich immunoassay	98/574	Not provided	0.009–80 ng/mL	Serum	
Beckmann-Coulter	Access Ultrasensitive hGH	Automated immunometric assay, Chemiluminescence	98/574	See comment	0.002–35 ng/mL (μ/L)	Serum or plasma (heparin)	Cross reaction analysed with GH 8 ng/mL for 20 K-GH: − 2542%
IDS	iSYS hGH	Chemilumminescent assay	98/574	22kD GH: 100%	0.05–100 ng/mL	Serum or plasma (heparin or EDTA)	Do not cross react with substances in these concentrations: 20 K-GH (10 ng/mL); placental GH (200 ng/mL); HPL (10,000 ng/mL); prolactin (40.000 ng/mL); pegvisomant (50,000 ng/mL); biotin 300 nmol/L); GHBP: 140 ng/mL. ng/mL × 3.0=μIU/mL
DIASource	hGH IRMA	Immunometric assay enzyme amplified sensitivity	98/574	Not provided	1–120 ng/mL Sensitivity: ng/mL	Serum, plasma	Conversion factor: 1μIU = 0.33 ng
	hGH EASIA	Enzyme-Immunoassay	98/574	Not provided	0.45–98 ng/mL Sensitivity:0.17 ng/mL	Serum, plasma	Conversion factor: 1μIU = 0.33 ng
CisBio	hGH-RIACT	Immunoradiometric	98/574	Not provided	0.03–75 ng/mL	Serum	1 ng = 3μIU; do not cross react with prolactin and hPL. Cross reaction with 20kD GH is less than 5% for the concentration up to 3750 ng/mL (22kD GH proportional concentration above 24,000 ng/mL)

### Data on clinical impact

The variability of GH secretion itself, the lack of a perfect correlation with other biochemical markers like IGF-I and other factors make the evaluation of disease amelioration and remission in GH related diseases challenging [62–65]. Problems with standardization or harmonization of GH assays add to the complexity and make the applicability of international guidelines difficult. Therefore, improvements in the assay agreement could help to permit comparability of published data and the clinical use of the information. For the clinician it is important to realize and understand the potential impact of assay problems on GH assay results to allow interpretation of local results in relation to published cut-offs and recommendations.

### Acromegaly

There are increasing data regarding the use of criteria for GH in the diagnosis of acromegaly [66]. Recommendations about the cut-off for GH during OGTT (oral glucose tolerance test) changed over time, with more recent publications recommending a nadir of 0.4 ng/mL or 1 ng/mL to exclude acromegaly and evaluate remission. In 2000, the “Cortina criteria” recommended a random GH concentration below 0.4 ng/mL or a GH nadir during OGTT below 1 ng/mL, together with normal IGF-I for age and sex [67]. After a decade, another consensus statements for “controlled acromegaly” recommended different GH cut-off values together with normal IGF-I. The recommendation stated a random GH concentration below 1 ng/mL and a GH nadir below 0.4 ng/mL. The current Endocrine Society Clinical Practice Guideline [68] requests the lack of suppression of GH below 1 ng/mL (and elevated IGF-I for age and sex) for diagnosing acromegaly. The suggested therapeutic goal was a random GH below 1 ng/mL coupled to a normal age-adjusted IGF-I. In part, the differences in the published cut-offs are related to differences in the assays used, although most guidelines do not specify the assay used to generate the GH concentrations stated. Some authors have published convincing evidence that prevalence of acromegaly as well as percentages for remission largely depend on cut-off values used [60, 61, 64, 65, 69, 70]. More recent publications on studies using modern, more specific GH assays calibrated against the latest recombinant standard clearly indicated that the above mentioned cut.-off of 1 ng/mL is inappropriately high and should be adapted for modern assays. The use of the “traditional” cut-off of 1 ng/mL can contribute to a delayed diagnosis in patients with milder forms of acromegaly. Using such assays, in cases of mild acromegaly GH can be suppressed to concentrations significantly below 1 ng/mL [65]. Apart from the fact that different assays have different sensitivity and therefore, different limits of quantification, it must be kept in mind that different assays also do measure different - though for most assays unspecified or unknown – subgroups of GH isoforms. Although the 22 K-GH is the most abundant and biological active isoform, in borderline cases the degree of specificity of the assay for the 22 K-GH or other isoforms could significantly affect the classification of the patient [71]. It is important to keep this in mind when applying cut-offs from guideline in such cases in clinical practice. A better standardization of GH assays is of great importance to facilitate diagnosis and to avoid misdiagnosis and insufficient treatment. In a complex disease like acromegaly undesirable consequences for patients have to be avoided. Uncertainties in monitoring the success of the sophisticated and expensive treatment options also have significant economic impacts to the health system.

Obviously, in addition to analytical factors, biological variables must also be taken into account when interpreting GH concentrations measured during the biochemical workup of suspected acromegaly. As an example, it has been proposed to adjust GH cut-offs for OGTT for sex and BMI to increase sensitivity of the test in the detection of acromegaly [72, 73]. Furthermore, the presence of renal failure can make the exclusion of acromegaly challenging: In these patients, high baseline GH levels are observed due to GH resistance – including an increase in the 20KD GH isoform [74]. The reduction in GH levels following oral glucose load might also be compromised. Although the literature is scarce, a case report on the exclusion of acromegaly in a patient with renal failure suggested that diagnosis must be made following dialysis: Baseline GH levels were lower compared to the situation before dialysis, and GH suppression during OGTT was normal [74].

### GH deficiency

Also in the diagnosis of GH deficiency biological variables like body mass index (BMI) can be important. Stimulation of GH by the insulin tolerance test might be affected only if BMI is greater than 35 kg/m^2^, but response to GHRH + arginine test generally has to be evaluated with cut-offs adjusted to BMI. Some authors advocate reducing the cut-off when the glucagon test is used in overweight/obese adults [69, 75]. Age and the stimulus used should also be considered as factors interfering with test interpretation [76, 77]. However, besides these interfering biological factors, it is important to recognize that different GH assays can reveal different GH concentrations in the same sample. Therefore, cut-offs need to be adjusted in an assay specific manner. Lower cut-off values are expected with the current recommended IS 98/574 when compared with the IS 80/505 [78], reflecting differences in isoform composition of the calibrators. Wagner et al. [76] analyzed samples from several stimulation tests in short children with and without GHD by distinct GH assays and proposed different cut-off values depending on the GH assay used. Such assay specific data are required for each GH assay used to allow an unbiased interpretation of the stimulation test outcome (Table 2). Using “general” cut-offs frequently quoted in guidelines with no reference for a specific assay is associated with the risk of misinterpretation, potentially leading to over- or under treatment.

**Table 2:** Cut-off limits derived from the same cohort of short children with different GH assays (adapted from reference [76])

Assay	Cut off limit (ng/mL)
Immulite 2000 (Siemens)	7.77
AutoDELFIA (Perkin-Elmer)	7.44
iSYS (IDS)	7.09
Liaison (DIASorin)	6.25
RIA (in-house Tübingen)	5.28
Dxl (Beckmann-Coulter)	5.15
ELISA (Mediagnost)	5.14
BC-IRMA (Beckmann-Coulter)	4.32

## Conclusion

GH is a complex and heterogeneous mixture of molecular isoforms and not a single, homogenous molecule. Although 22 K-GH is the main isoform in circulation, there are other GH isoforms that can represent approximately 10–20% of GH under physiological conditions. Usually, GH isoforms are secreted in parallel in response to various stimuli, with changes in 22 K-GH mirroring changes in all isoforms. Some studies have reported minor changes in the non-22 K/22 K-GH and 22 K/20 K-GH ratio under certain conditions, mostly with elevated abundance of the non-22 K isoforms. Such variation might be due to differences in the half-life of the isoforms, but might also be related to limitations in current assay methods to accurately quantify the various isoforms over a wide concentration range. More studies are needed to better understand why some diseases including pituitary adenomas might lead to changes in the isoform ratios, and to evaluate if changes in isoforms might be of clinical relevance. More importantly in clinical routine, the existence of GH isoforms represents one of the main reasons for discrepancies in GH concentrations measured by different common GH assays. Efforts to harmonize GH assays are under way but it remains important for the clinician to understand the potential impact of the specific GH assay used on the GH concentrations reported. Clinical decision limits and cut-off values not only must be adapted to biological variables, but also to the specific GH assay used by the local laboratory.
